# Crystal structure of ethyl 3-anilino-2-{[bis­(methyl­sulfan­yl)methyl­idene]amino}-3-oxopropano­ate

**DOI:** 10.1107/S1600536814016560

**Published:** 2014-08-01

**Authors:** A. Kémish López-Rodríguez, Alfonso Lira-Rocha, Marcos Flores-Alamo

**Affiliations:** a Departamento de Farmacia, Facultad de Química, Universidad Nacional Autónoma de México, 04510, México DF, Mexico; b Facultad de Química, Universidad Nacional Autónoma de México, 04510, México DF, Mexico

**Keywords:** crystal structure, thia­zolo[5,4-*b*]quinoline derivative, hydrogen bonding

## Abstract

The mol­ecular conformation of the title compound, C_14_H_18_N_2_O_3_S_2_, is stabilized by intra­molecular N—H⋯N and C—H⋯O hydrogen bonds. The crystal packing is characterized by a series of C—H⋯O hydrogen bonds, resulting in a three-dimensional network.

## Related literature   

For the synthesis and cytotoxic activity of thia­zolo[5,4-*b*]quinoline derivatives, see: Rodríguez-Loaiza *et al.* (2004 ▶); Loza-Mejía *et al.* (2008 ▶, 2009 ▶); Adams *et al.* (2002 ▶).
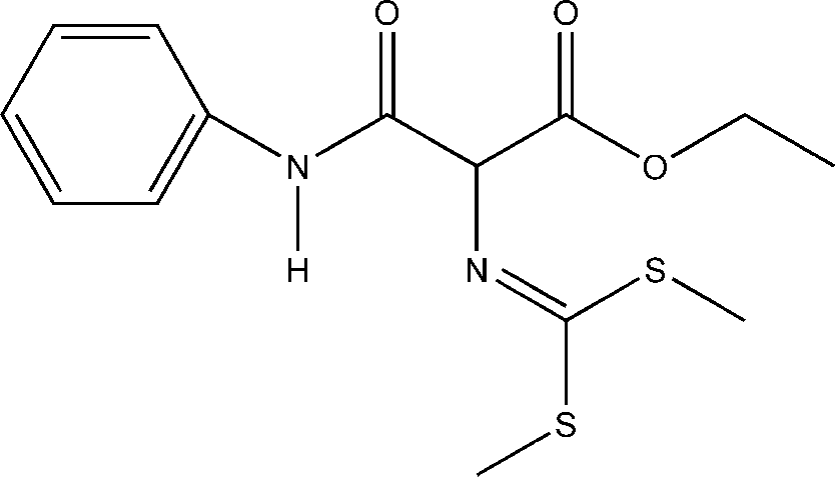


## Experimental   

### Crystal data  

C_14_H_18_N_2_O_3_S_2_
*M* *_r_* = 326.42
Triclinic, 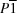

*a* = 8.5298 (11) Å
*b* = 9.1422 (16) Å
*c* = 11.0268 (13) Å
α = 101.377 (12)°
β = 102.102 (10)°
γ = 104.457 (13)°
*V* = 785.3 (2) Å^3^
*Z* = 2
Mo *K*α radiation
μ = 0.35 mm^−1^
*T* = 145 K
0.6 × 0.5 × 0.35 mm

### Data collection  

Agilent Xcalibur Atlas Gemini diffractometer
Absorption correction: analytical (*CrysAlis RED*; Agilent, 2012 ▶) *T*_min_ = 0.87, *T*_max_ = 0.922
5879 measured reflections
3625 independent reflections
3022 reflections with *I* > 2σ(*I*)
*R*_int_ = 0.028

### Refinement  

*R*[*F* ^2^ > 2σ(*F* ^2^)] = 0.039
*wR*(*F* ^2^) = 0.101
*S* = 1.05
3625 reflections
197 parameters
1 restraint
H atoms treated by a mixture of independent and constrained refinement
Δρ_max_ = 0.34 e Å^−3^
Δρ_min_ = −0.36 e Å^−3^

### 

Data collection: *CrysAlis PRO* (Agilent, 2012 ▶); cell refinement: *CrysAlis CCD*; data reduction: *CrysAlis RED* (Agilent, 2012 ▶); program(s) used to solve structure: *SHELXS97* (Sheldrick, 2008 ▶); program(s) used to refine structure: *SHELXL97* (Sheldrick, 2008 ▶); molecular graphics: *Mercury* (Macrae *et al.*, 2006 ▶); software used to prepare material for publication: *WinGX* (Farrugia, 2012 ▶).

## Supplementary Material

Crystal structure: contains datablock(s) global, I. DOI: 10.1107/S1600536814016560/bt6988sup1.cif

Structure factors: contains datablock(s) I. DOI: 10.1107/S1600536814016560/bt6988Isup2.hkl

Supporting information file. DOI: 10.1107/S1600536814016560/bt6988Isup3.cml

**Fig. 1.**
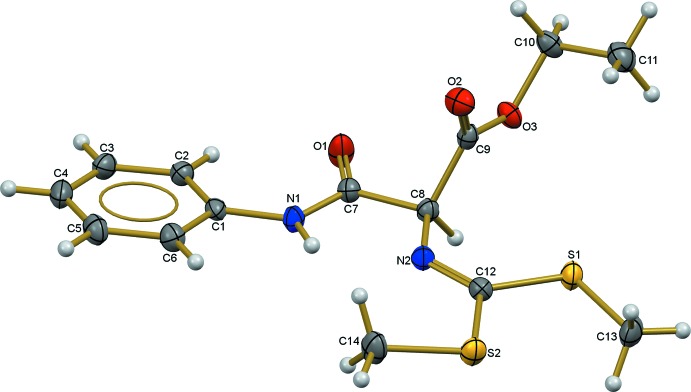
The molecular structure of the title compound. Displacement ellipsoids are drawn at the 50% probability level and H atoms are shown as circles of arbitrary size.

. DOI: 10.1107/S1600536814016560/bt6988fig1.tif
The mol­ecular structure of the title compound. Displacement ellipsoids are drawn at the 50% probability level and H atoms are shown as circles of arbitrary size.

**Fig. 2.**
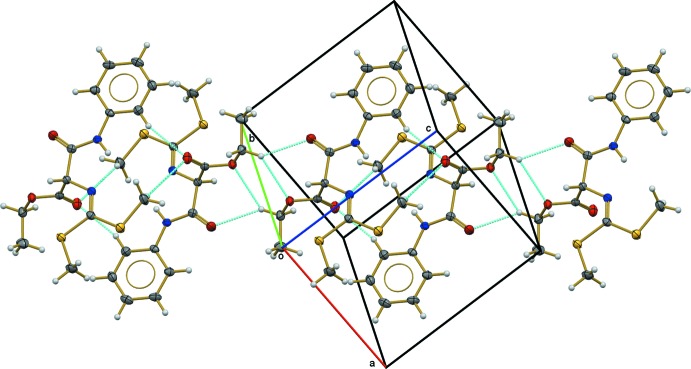
Crystal packing with intermolecular interactions of type C—H···O forming a three-dimensional network.

. DOI: 10.1107/S1600536814016560/bt6988fig2.tif
Crystal packing with inter­molecular inter­actions of type C—H⋯O forming a three-dimensional network.

CCDC reference: 1014381

Additional supporting information:  crystallographic information; 3D view; checkCIF report

## Figures and Tables

**Table 1:** Hydrogen-bond geometry (Å, °)

*D*—H⋯*A*	*D*—H	H⋯*A*	*D*⋯*A*	*D*—H⋯*A*
N1—H1*F*⋯N2	0.889 (15)	2.019 (18)	2.586 (2)	120.5 (15)
C2—H2⋯O1	0.95	2.33	2.932 (2)	121
C6—H6⋯O2^i^	0.95	2.4	3.295 (2)	156
C10—H10*B*⋯O1^ii^	0.99	2.53	3.340 (2)	138
C10—H10*B*⋯O3^ii^	0.99	2.65	3.377 (2)	131
C11—H11*A*⋯O2^iii^	0.98	2.64	3.465 (2)	141
C13—H13*B*⋯O1^iv^	0.98	2.63	3.579 (2)	162

Symmetry codes: (i) 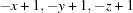; (ii) 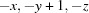; (iii) 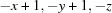; (iv) 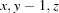.

## supplementary crystallographic information

### S1. Comment

Quinoline fused five-membered heterocyclic compounds have been the subject of sustained interest because many of them have cytotoxic properties. Thus, they are potential antitumor agents (Adams *et al.*, 2002). We have reported the synthesis and cytotoxic activity of several thiazolo[5,4-*b*]quinoline (TQ) derivatives (Rodríguez-Loaiza *et al.*, 2004; Loza-Mejía *et al.*, 2008; Loza-Mejía *et al.*, 2009.). During a study on the synthesis of new oxazolo[5,4-*b*]quinoline derivatives, which can be considered as analogues of TQ, the preparation of a key intermediate was tried by using a procedure previously reported by our group.

In the title compound, the asymmetric unit consist of one molecule of the ethyl-2-{[bis(methylsulfanyl)methylidene]amino}-3-oxo-3-(phenylamino)-propanoate (Fig. 1). The planes formed by phenyl ring C1/C6 (equation plane: 6.499 (4) *x* + 3.679 (6) *y* - 5.701 (6) *z* = 0.613 (6)) and the N1—C7/O1—C8 group (equation plane: 6.941 (4) *x* + 3.217 (7) *y* - 4.499 (8) *z* = 1.040 (4)) are almost coplanar with a dihedral angle between them of 7.64 (11)°; of the same way the dihedral angle of 8.34 (9)° between planes formed by N1—C7/O1—C8 and S1—C12/N2—S2 (equation plane: 7.465 (2) *x* + 2.049 (5) *y* - 4.965 (2) *z* = 0.633 (9)) evidence the coplanarity. On the other hand, the plane formed by C8—C9/O2—O3 (equation plane: - 3.115 (6) *x* + 8.551 (3) *y* + 1.760 (9) *z* = 3.314 (1)) shows a behavior near to orthogonality with the other planes.

In the crystal structure there are intermolecular C—H···O contacts (Table 1) connecting the molecules to a three-dimensional network.

### S2. Experimental

Ethyl {[bis(methylsulfanyl)methylidene]amino}acetate was reacted with phenyl isocyanate at low temperature (-75°) under basic conditions, in order to obtain the oxazole derivative which is a intermediate suitable for the formation of the oxazolo[5,4-*b*]quinoline system. Surprisingly, this reaction gave in a high yield a different crystal intermediate, whose structure was characterized by IR, NMR and X-ray studies. Yield: 66.7%. Colorless crystals; mp: 103°C; IR (*ν*max, cm^-1^): 3283 (–NH amidic); 2982, 2930, 2891 (–CH aliph.); 1733 (C=O ester); 1687 (C=O amidic); 1H NMR (400 MHz, DMSO-*d _6_*): d 1.17 (t, *J* = 7.1 Hz, 3H) –CH_3_; 2.46 (s, 3H) –SCH_3_; 2.59 (s, 3H) –SCH_3_; 4.14 (q, *J* = 7.1, 2H) –CH_2_; 5.00 (s, 1H) –CH; 7.07 (t, *J* = 7.8 Hz, 1H) –H4; 7.31 (d, *J* = 7.8 Hz, 2H) –H3, –H5; 7.61 (d, J = 8.4 Hz, 2H) –H2, –H6; 9.97 (s, 1H) –NH–; ^13^C NMR (101 MHz, DMSO– *d _6_*): d 14.42, 14.98, 15.26, 61.69, 69.68, 120.03, 124.33, 129.20, 138.74, 165.34, 167.06.

### S3. Refinement

The H atom of the amine group (N1/H1F) was located in a difference map and refined isotropically with *U*_iso_(H) = 1.2*U*_eq_(N). The N-H distance was restrained to 0.92 (2)Å. H atoms attached to C atoms were placed in geometrically idealized positions and refined as riding on their parent atoms, with C—H = 0.95–0.99 Å and with *U*_iso_(H) = 1.2*U*_eq_(C), for aromatic and methylene groups and *U*_iso_(H) = 1.5*U*_e_q(C) for methyl groups.

### Crystal data

**Table:**

C_14_H_18_N_2_O_3_S_2_	*Z* = 2
*M**_r_* = 326.42	*F*(000) = 344
Triclinic, *P*1	*D*_x_ = 1.38 Mg m^−^^3^
Hall symbol: -P 1	Mo *K*α radiation, λ = 0.71073 Å
*a* = 8.5298 (11) Å	Cell parameters from 2290 reflections
*b* = 9.1422 (16) Å	θ = 3.6–29.4°
*c* = 11.0268 (13) Å	µ = 0.35 mm^−^^1^
α = 101.377 (12)°	*T* = 145 K
β = 102.102 (10)°	Block, colourless
γ = 104.457 (13)°	0.6 × 0.5 × 0.35 mm
*V* = 785.3 (2) Å^3^	

### Data collection

**Table:**

Agilent Xcalibur Atlas Gemini diffractometer	3625 independent reflections
Graphite monochromator	3022 reflections with *I* > 2σ(*I*)
Detector resolution: 10.4685 pixels mm^-1^	*R*_int_ = 0.028
ω scans	θ_max_ = 29.4°, θ_min_ = 3.6°
Absorption correction: analytical (*CrysAlis RED*; Agilent, 2012)	*h* = −11→11
*T*_min_ = 0.87, *T*_max_ = 0.922	*k* = −9→11
5879 measured reflections	*l* = −15→14

### Refinement

**Table:**

Refinement on *F*^2^	H atoms treated by a mixture of independent and constrained refinement
Least-squares matrix: full	*w* = 1/[σ^2^(*F*_o_^2^) + (0.0448*P*)^2^ + 0.1551*P*] where *P* = (*F*_o_^2^ + 2*F*_c_^2^)/3
*R*[*F*^2^ > 2σ(*F*^2^)] = 0.039	(Δ/σ)_max_ < 0.001
*wR*(*F*^2^) = 0.101	Δρ_max_ = 0.34 e Å^−^^3^
*S* = 1.05	Δρ_min_ = −0.36 e Å^−^^3^
3625 reflections	Extinction correction: *SHELXL97* (Sheldrick, 2008), Fc^*^=kFc[1+0.001xFc^2^λ^3^/sin(2θ)]^-1/4^
197 parameters	Extinction coefficient: 0.033 (3)
1 restraint	

### Special details

**Table:**

Geometry. All e.s.d.'s (except the e.s.d. in the dihedral angle between two l.s. planes) are estimated using the full covariance matrix. The cell e.s.d.'s are taken into account individually in the estimation of e.s.d.'s in distances, angles and torsion angles; correlations between e.s.d.'s in cell parameters are only used when they are defined by crystal symmetry. An approximate (isotropic) treatment of cell e.s.d.'s is used for estimating e.s.d.'s involving l.s. planes.

### Fractional atomic coordinates and isotropic or equivalent isotropic displacement parameters (Å^2^)

**Table:**

	*x*	*y*	*z*	*U*_iso_*/*U*_eq_	
C1	0.22485 (19)	0.68715 (19)	0.59186 (14)	0.0184 (3)	
C2	0.1478 (2)	0.8032 (2)	0.57936 (16)	0.0216 (4)	
H2	0.0786	0.7997	0.4985	0.026*	
C3	0.1738 (2)	0.9243 (2)	0.68710 (16)	0.0260 (4)	
H3	0.1217	1.0038	0.6792	0.031*	
C4	0.2741 (2)	0.9311 (2)	0.80562 (17)	0.0289 (4)	
H4	0.2909	1.0146	0.8784	0.035*	
C5	0.3498 (2)	0.8150 (2)	0.81709 (16)	0.0276 (4)	
H5	0.4188	0.819	0.8981	0.033*	
C6	0.3254 (2)	0.6930 (2)	0.71085 (16)	0.0241 (4)	
H6	0.3773	0.6135	0.7193	0.029*	
C7	0.13582 (19)	0.5349 (2)	0.36177 (15)	0.0194 (3)	
C8	0.1513 (2)	0.38584 (19)	0.27795 (14)	0.0187 (3)	
H8	0.0362	0.3141	0.2294	0.022*	
C9	0.24576 (19)	0.43850 (19)	0.18264 (15)	0.0188 (3)	
C10	0.2175 (2)	0.4733 (2)	−0.02788 (15)	0.0244 (4)	
H10A	0.3176	0.5663	0.0132	0.029*	
H10B	0.1363	0.5023	−0.0897	0.029*	
C11	0.2686 (2)	0.3427 (2)	−0.09767 (18)	0.0325 (4)	
H11A	0.315	0.3746	−0.1659	0.049*	
H11B	0.1701	0.2496	−0.1356	0.049*	
H11C	0.3541	0.3186	−0.0372	0.049*	
C12	0.2467 (2)	0.1698 (2)	0.31142 (14)	0.0195 (3)	
C13	0.2088 (2)	−0.1139 (2)	0.13307 (17)	0.0294 (4)	
H13A	0.1576	−0.18	0.0448	0.044*	
H13B	0.1628	−0.1673	0.1925	0.044*	
H13C	0.3309	−0.0944	0.1545	0.044*	
C14	0.4017 (3)	0.2120 (2)	0.56368 (16)	0.0317 (4)	
H14A	0.4778	0.3092	0.5589	0.048*	
H14B	0.4595	0.1723	0.6305	0.048*	
H14C	0.3018	0.2324	0.5847	0.048*	
N1	0.20693 (17)	0.56002 (17)	0.48823 (12)	0.0200 (3)	
N2	0.23886 (17)	0.30580 (16)	0.35854 (12)	0.0200 (3)	
O1	0.06584 (17)	0.61847 (16)	0.31231 (11)	0.0308 (3)	
O2	0.39713 (14)	0.48975 (15)	0.20888 (11)	0.0265 (3)	
O3	0.14030 (13)	0.42455 (14)	0.07059 (10)	0.0217 (3)	
S1	0.16283 (5)	0.06980 (5)	0.14655 (4)	0.02388 (13)	
S2	0.33924 (6)	0.06874 (5)	0.41135 (4)	0.02561 (14)	
H1F	0.254 (2)	0.487 (2)	0.5037 (18)	0.031*	

### Atomic displacement parameters (Å^2^)

**Table:**

	*U*^11^	*U*^22^	*U*^33^	*U*^12^	*U*^13^	*U*^23^
C1	0.0190 (7)	0.0190 (9)	0.0168 (7)	0.0042 (6)	0.0079 (6)	0.0029 (6)
C2	0.0246 (8)	0.0205 (9)	0.0218 (8)	0.0075 (7)	0.0089 (7)	0.0064 (7)
C3	0.0335 (9)	0.0187 (9)	0.0300 (9)	0.0100 (7)	0.0155 (8)	0.0061 (7)
C4	0.0343 (9)	0.0227 (10)	0.0251 (9)	0.0037 (8)	0.0121 (8)	−0.0017 (7)
C5	0.0272 (9)	0.0317 (11)	0.0187 (8)	0.0059 (8)	0.0030 (7)	0.0024 (7)
C6	0.0243 (8)	0.0268 (10)	0.0220 (8)	0.0095 (7)	0.0061 (7)	0.0059 (7)
C7	0.0205 (7)	0.0190 (9)	0.0189 (7)	0.0062 (6)	0.0062 (7)	0.0044 (6)
C8	0.0210 (7)	0.0178 (8)	0.0165 (7)	0.0065 (6)	0.0035 (6)	0.0040 (6)
C9	0.0217 (8)	0.0162 (8)	0.0182 (7)	0.0080 (6)	0.0036 (7)	0.0025 (6)
C10	0.0272 (8)	0.0315 (10)	0.0191 (8)	0.0124 (7)	0.0075 (7)	0.0113 (7)
C11	0.0356 (10)	0.0377 (12)	0.0288 (9)	0.0133 (9)	0.0161 (8)	0.0086 (8)
C12	0.0212 (7)	0.0201 (9)	0.0177 (7)	0.0057 (6)	0.0062 (7)	0.0055 (6)
C13	0.0382 (10)	0.0196 (9)	0.0280 (9)	0.0107 (8)	0.0077 (8)	−0.0001 (7)
C14	0.0426 (10)	0.0306 (11)	0.0177 (8)	0.0121 (9)	−0.0001 (8)	0.0043 (7)
N1	0.0251 (7)	0.0197 (8)	0.0175 (6)	0.0116 (6)	0.0057 (6)	0.0040 (5)
N2	0.0243 (7)	0.0189 (7)	0.0173 (6)	0.0082 (6)	0.0048 (6)	0.0049 (5)
O1	0.0461 (7)	0.0291 (7)	0.0208 (6)	0.0229 (6)	0.0034 (6)	0.0061 (5)
O2	0.0202 (6)	0.0324 (8)	0.0245 (6)	0.0052 (5)	0.0029 (5)	0.0092 (5)
O3	0.0208 (6)	0.0300 (7)	0.0153 (5)	0.0094 (5)	0.0038 (5)	0.0072 (5)
S1	0.0304 (2)	0.0213 (2)	0.0173 (2)	0.00912 (18)	0.00318 (17)	0.00107 (16)
S2	0.0351 (3)	0.0212 (2)	0.0211 (2)	0.01201 (19)	0.00365 (19)	0.00657 (17)

### Geometric parameters (Å, º)

**Table:**

C1—C6	1.392 (2)	C10—O3	1.4683 (19)
C1—C2	1.395 (2)	C10—C11	1.498 (3)
C1—N1	1.412 (2)	C10—H10A	0.99
C2—C3	1.390 (2)	C10—H10B	0.99
C2—H2	0.95	C11—H11A	0.98
C3—C4	1.384 (3)	C11—H11B	0.98
C3—H3	0.95	C11—H11C	0.98
C4—C5	1.386 (3)	C12—N2	1.273 (2)
C4—H4	0.95	C12—S2	1.7587 (17)
C5—C6	1.388 (2)	C12—S1	1.7678 (16)
C5—H5	0.95	C13—S1	1.8035 (19)
C6—H6	0.95	C13—H13A	0.98
C7—O1	1.2217 (19)	C13—H13B	0.98
C7—N1	1.347 (2)	C13—H13C	0.98
C7—C8	1.541 (2)	C14—S2	1.7968 (18)
C8—N2	1.461 (2)	C14—H14A	0.98
C8—C9	1.532 (2)	C14—H14B	0.98
C8—H8	1	C14—H14C	0.98
C9—O2	1.2075 (19)	N1—H1F	0.889 (15)
C9—O3	1.3292 (18)		
			
C6—C1—C2	120.11 (15)	C11—C10—H10A	109.6
C6—C1—N1	116.63 (15)	O3—C10—H10B	109.6
C2—C1—N1	123.25 (14)	C11—C10—H10B	109.6
C3—C2—C1	118.97 (16)	H10A—C10—H10B	108.2
C3—C2—H2	120.5	C10—C11—H11A	109.5
C1—C2—H2	120.5	C10—C11—H11B	109.5
C4—C3—C2	121.22 (17)	H11A—C11—H11B	109.5
C4—C3—H3	119.4	C10—C11—H11C	109.5
C2—C3—H3	119.4	H11A—C11—H11C	109.5
C3—C4—C5	119.36 (16)	H11B—C11—H11C	109.5
C3—C4—H4	120.3	N2—C12—S2	120.47 (12)
C5—C4—H4	120.3	N2—C12—S1	123.28 (12)
C4—C5—C6	120.39 (16)	S2—C12—S1	116.23 (10)
C4—C5—H5	119.8	S1—C13—H13A	109.5
C6—C5—H5	119.8	S1—C13—H13B	109.5
C5—C6—C1	119.94 (17)	H13A—C13—H13B	109.5
C5—C6—H6	120	S1—C13—H13C	109.5
C1—C6—H6	120	H13A—C13—H13C	109.5
O1—C7—N1	126.13 (16)	H13B—C13—H13C	109.5
O1—C7—C8	120.39 (14)	S2—C14—H14A	109.5
N1—C7—C8	113.48 (14)	S2—C14—H14B	109.5
N2—C8—C9	112.30 (13)	H14A—C14—H14B	109.5
N2—C8—C7	110.24 (12)	S2—C14—H14C	109.5
C9—C8—C7	106.50 (13)	H14A—C14—H14C	109.5
N2—C8—H8	109.2	H14B—C14—H14C	109.5
C9—C8—H8	109.2	C7—N1—C1	129.37 (14)
C7—C8—H8	109.2	C7—N1—H1F	111.8 (13)
O2—C9—O3	125.06 (15)	C1—N1—H1F	118.7 (13)
O2—C9—C8	123.37 (14)	C12—N2—C8	120.84 (13)
O3—C9—C8	111.54 (13)	C9—O3—C10	116.15 (12)
O3—C10—C11	110.09 (15)	C12—S1—C13	104.78 (8)
O3—C10—H10A	109.6	C12—S2—C14	99.96 (8)
			
C6—C1—C2—C3	0.3 (2)	O1—C7—N1—C1	−2.9 (3)
N1—C1—C2—C3	−179.69 (15)	C8—C7—N1—C1	176.35 (15)
C1—C2—C3—C4	−0.1 (3)	C6—C1—N1—C7	−171.05 (16)
C2—C3—C4—C5	−0.1 (3)	C2—C1—N1—C7	8.9 (3)
C3—C4—C5—C6	0.0 (3)	S2—C12—N2—C8	−175.45 (11)
C4—C5—C6—C1	0.2 (3)	S1—C12—N2—C8	2.8 (2)
C2—C1—C6—C5	−0.4 (2)	C9—C8—N2—C12	−70.50 (19)
N1—C1—C6—C5	179.60 (15)	C7—C8—N2—C12	170.92 (14)
O1—C7—C8—N2	−179.82 (15)	O2—C9—O3—C10	1.2 (2)
N1—C7—C8—N2	0.92 (19)	C8—C9—O3—C10	179.46 (13)
O1—C7—C8—C9	58.11 (19)	C11—C10—O3—C9	83.60 (18)
N1—C7—C8—C9	−121.15 (14)	N2—C12—S1—C13	179.81 (14)
N2—C8—C9—O2	−38.3 (2)	S2—C12—S1—C13	−1.86 (12)
C7—C8—C9—O2	82.41 (19)	N2—C12—S2—C14	−1.14 (16)
N2—C8—C9—O3	143.40 (13)	S1—C12—S2—C14	−179.52 (10)
C7—C8—C9—O3	−95.84 (15)		

### Hydrogen-bond geometry (Å, º)

**Table:**

*D*—H···*A*	*D*—H	H···*A*	*D*···*A*	*D*—H···*A*
N1—H1*F*···N2	0.889 (15)	2.019 (18)	2.586 (2)	120.5 (15)
C2—H2···O1	0.95	2.33	2.932 (2)	121
C6—H6···O2^i^	0.95	2.4	3.295 (2)	156
C10—H10*B*···O1^ii^	0.99	2.53	3.340 (2)	138
C10—H10*B*···O3^ii^	0.99	2.65	3.377 (2)	131
C11—H11*A*···O2^iii^	0.98	2.64	3.465 (2)	141
C13—H13*B*···O1^iv^	0.98	2.63	3.579 (2)	162

Symmetry codes: (i) −*x*+1, −*y*+1, −*z*+1; (ii) −*x*, −*y*+1, −*z*; (iii) −*x*+1, −*y*+1, −*z*; (iv) *x*, *y*−1, *z*.
