# Supplementary figures and images for: Crystal structure of ethyl 3-anilino-2-{[bis­(methyl­sulfan­yl)methyl­idene]amino}-3-oxopropano­ate
Source: Acta Crystallogr Sect E Struct Rep Online. 2014 Aug 1;70(Pt 9):o930–1. doi: 10.1107/S1600536814016560 (PMC4186088; doi:10.1107/S1600536814016560)

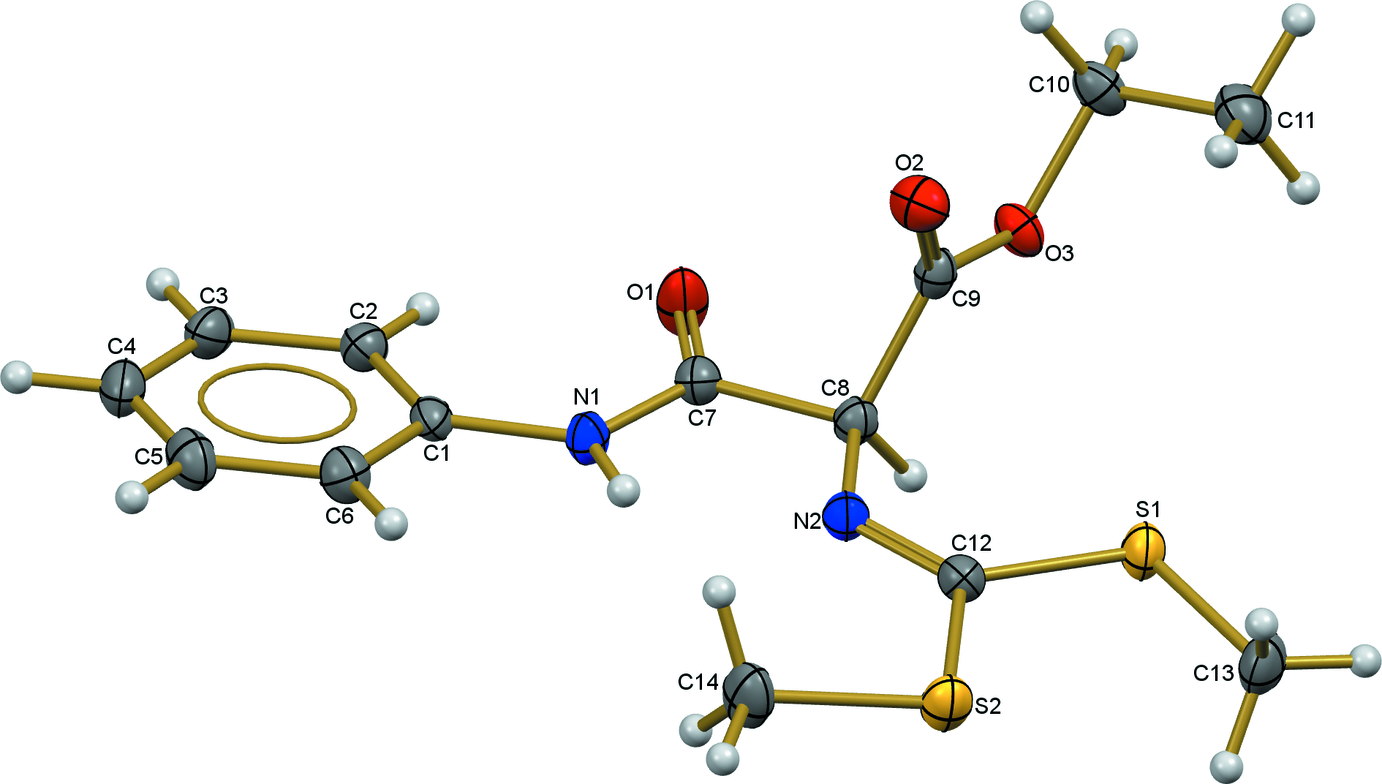

Supplement: Supplementary file 4 [file e-70-0o930-fig1.tif]

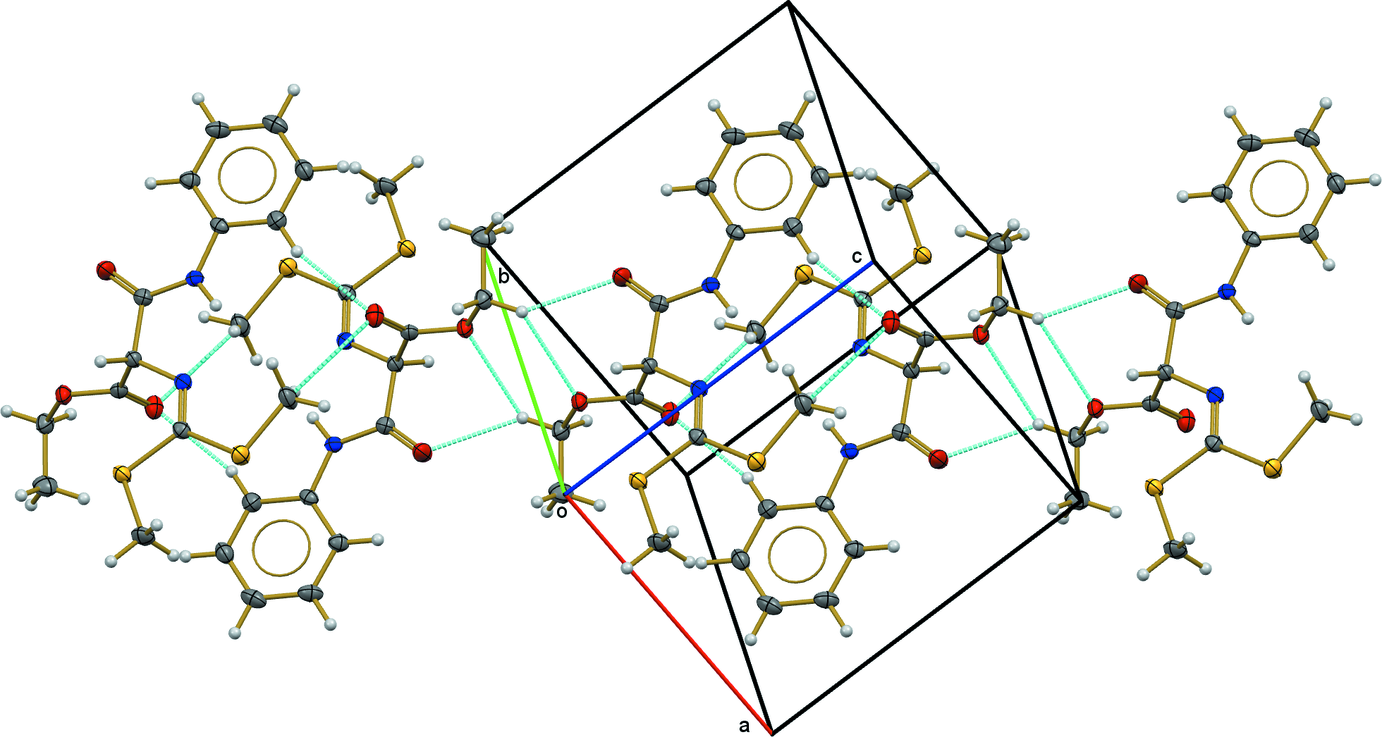

Supplement: Supplementary file 5 [file e-70-0o930-fig2.tif]
